# Educational Intervention to Increase COVID-19 Vaccine Uptake in Rural Patients with Chronic Diseases: Lessons Learned from An Innovative Academic–Community Partnership

**DOI:** 10.3390/ijerph21010071

**Published:** 2024-01-08

**Authors:** Ranjita Misra, Brenna Kirk, Samantha Shawley-Brzoska, Daniel Totzkay, Catherine Morton, Summer Kuhn, Misty Harris, Mary McMillion, Elaine Darling

**Affiliations:** 1 West Virginia University School of Public Health, Morgantown, WV 26505, USA; bok0001@mix.wvu.edu (B.K.); sshawley@hsc.wvu.edu (S.S.-B.); 2 Department of Communication Studies, Eberly College of Art and Sciences, West Virginia University, Morgantown, WV 26506, USA; daniel.totzkay@mail.wvu.edu; 3 Health Sciences and Technology Academy, Morgantown, WV 26506, USA; catherine.morton@hsc.wvu.edu (C.M.); summer.kuhn@hsc.wvu.edu (S.K.); miharris1@hsc.wvu.edu (M.H.); mlmcmillion@hsc.wvu.edu (M.M.); 4 The Center for Rural Health Development Inc., 75 Chase Dr, Hurricane, WV 25526, USA; elaine.darling@wvruralhealth.org

**Keywords:** implementation, rural adults, chronic condition, COVID-19 vaccine uptake, health navigator

## Abstract

Background: The pandemic has disproportionately impacted rural communities with a higher burden of chronic disease and COVID-19 infection. West Virginia is a rural state with a high rate of diabetes, hypertension, and COPD, which are known risk factors for severe COVID-19 and long COVID. Yet, there is a significant hesitancy regarding COVID-19 vaccination uptake in the state. The purpose of this study was to use an educational intervention to increase vaccine knowledge and vaccine acceptance in rural patients with chronic disease(s) in West Virginia. This project used an academic–community partnership comprised of researchers, practitioners, community organizations, community-engaged partners, and patient stakeholders to increase COVID-19 health literacy and increase vaccine acceptance among rural West Virginians with chronic conditions. Materials and Methods: A quasi-experimental study design was used to deliver an educational intervention by trained Health Navigators using short videos to increase COVID-19 health literacy and address participants’ vaccine concerns. Eligibility included adults (18 years and older) who have at least one chronic condition. A statewide community advisory board (CAB) guided the development of the educational training curriculum and implementation strategies. An adapted version of the Exploration, Preparation, Implementation, Sustainment (EPIS) framework guided the development of the intervention. Health Navigators (*n* = 45) delivered the educational intervention in their local communities between November 2022 and October 2023 (project implementation is still ongoing). Intervention fidelity checks, an adaptable script, and a flow chart allowed tailoring of brief videos to address participants’ specific COVID-19 questions and vaccine concerns. A validated online survey, monitored by an online Research Electronic Data Capture (REDCap) database, assessed participants’ knowledge, perceived susceptibility, and vaccine intention. Results: Health Navigators delivered the intervention to 1368 West Virginians in 52 counties (59.2% women; 61.8% without a college degree). Participants reported living with an average of 2.1 ± 1.4 chronic conditions. The mean age was 43.5 ± 18.8 years. The majority of participants (81.2%) had received the primary vaccination series, and 63.1% had at least one booster. However, 18% were unvaccinated or did not complete the primary COVID-19 vaccine series. Discussions to improve vaccine literacy focused on how the vaccine was so quickly developed and protects against variants, addressing concerns related to the safety, short- and long-term side effects, and importance of vaccine uptake for immunocompromised individuals. Participants with higher concerns were more likely to be unvaccinated and to have not completed their primary series or boosters (*p* < 0.001). However, the educational intervention improved the willingness of individuals who were either unvaccinated or did not complete their primary vaccine series to get vaccinated (11.4%). Discussion: Our findings highlight the importance of vaccine literacy in increasing vaccination rates among rural patients with chronic diseases. Using the EPIS framework allowed us to reflect upon the challenges, ensure resilience during changing local contexts, and plan and implement a promising, cost-effective intervention in rural areas. Conclusions: This study provides insights into the need for tailored educational interventions based on disease status, which has implications for public health and patient care in rural and underserved communities. Academic–community partnerships can be useful for successful knowledge transfer for vaccine acceptance to reduce rural health disparities.

## 1. Introduction

The COVID-19 pandemic is a global health crisis that has disproportionately impacted individuals living with chronic conditions [1,2]. Chronic diseases have long been used as key indicators of health, and their role in poor COVID-19 outcomes has been particularly pronounced. Diabetes, chronic lung conditions, and heart disease have emerged as increasing risks of adverse outcomes (such as hospitalization, ventilation, and intensive care) and even death from severe COVID-19 illness [3]. Other factors that are associated with greater risk for COVID-19-related complications include older age [4], belonging to a racial or ethnic minority group [5,6], and living in areas that are rural and/or socially vulnerable [7,8,9].

West Virginia (WV) is the third most rural state in the United States (US) and has a substantial chronic disease burden, making its population especially vulnerable to COVID-19 illness and related complications. In fact, WV has the highest rates of diabetes (16.2%), cardiovascular disease (15.5%), chronic obstructive pulmonary disease (COPD; 15.3%), kidney disease (4.6%), and depression (26.6%) in the US [10]. In addition to high rates of chronic disease, WV is impacted by various social determinants of health, such as high rates of poverty, lower educational attainment, low health literacy, and a high prevalence of health professional shortage areas [11,12]. The culmination of these factors contributed to WV having the fourth-highest COVID-19-related mortality rate in the U.S. in 2021 (146.8 per 100,000) [13]. Furthermore, West Virginia has disproportionately experienced greater rates of long COVID incidence (18.2% vs. 14.0% nationally in 2022) [14]. Despite West Virginian’s increased vulnerability to COVID-19 and its related complications, unique challenges in the distribution and acceptance of COVID-19 vaccinations remain in the state.

To date, vaccination against the virus that causes COVID-19 and its variants remains one of the most effective ways to protect individuals against COVID-19 infection, complications, and subsequent long COVID conditions [15]. However, vaccine hesitancy remains a critical barrier to global vaccination uptake [16]. While vaccine hesitancy has been observed in various contexts, the COVID-19 pandemic has brought it to the forefront of public health discourse. Vaccine hesitancy is a phenomenon characterized by reluctance or refusal to be vaccinated, and reported factors contributing to this hesitancy include concerns about vaccine safety and efficacy, as well as misinformation and mistrust in the healthcare system [17].

COVID-19 vaccines are now more widely available and accessible to individuals living in rural areas of WV, though some are still reluctant to be vaccinated or have no plans to be vaccinated. As of May 2023, only 77.8% of West Virginian adults have had at least one dose of a COVID-19 vaccine compared to 92.3% nationally [18]. Furthermore, only 14.3% of adults living in WV have had an updated (bivalent) COVID-19 booster dose compared to 20.5% nationally [18]. Within the context of WV’s high chronic disease burden, aging population, and poor social determinants of health (SDoH) factors affecting health outcomes in the state, it is clear that a significant gap remains in COVID-19 prevention and vaccination uptake.

As the world continues to navigate the COVID-19 pandemic, understanding the interplay between chronic disease, vaccine hesitancy, and regional disparities is essential. Especially within the context of WV, innovative educational interventions and strategies to increase vaccine intentions and uptake behavior, as well as address vaccine concerns and hesitancy, are needed [19]. Therefore, the purpose of this study was to assess the impact of a community-based educational intervention to increase COVID-19 health literacy and vaccine acceptance in rural patients with chronic diseases. The project used an innovative academic–community partnership to guide the planning and implementation of the project, and trained Health Navigators delivered a brief educational intervention in their local West Virginia communities.

## 2. Materials and Methods

### 2.1. Academic–Community Partnership

The multidisciplinary core project team represented many disciplines (public health practice, health education, chronic disease prevention, health services research, and health communication) within the academic community to increase the capacity to serve chronic disease patients in rural and underserved communities and enhance translation of evidence-based knowledge to real-world settings. The academic–community partnership comprises researchers, practitioners, community organizations, community-engaged partners, and patient stakeholders. Building on previously established partnerships and developing new opportunities for collaboration, an innovative academic–community partnership was formed to develop, implement, and evaluate the current project. One major collaborator was the WV Health Sciences and Technology Academy (HSTA), a well-established mentoring program in the state that aims to help underrepresented high school students pursue STEM-related undergraduate and graduate education. Partnering with WV HSTA allowed for the recruitment of junior and senior high school students in tandem with undergraduate and graduate college students to be trained as Health Navigators (HNs) to deliver the intervention in their local communities throughout the state. Lastly, engaging Health Navigators (high school and college students from local areas) in this project allowed students to gain experiential learning opportunities in public health practice.

An additional partner that was crucial to the development of the educational intervention was the Center for Rural Health Development (CRHD), a nonprofit organization with the mission to improve the health of West Virginians and strengthen West Virginia’s health care delivery system, especially in rural communities. The center serves as the lead agency for the West Virginia Immunization Network, a statewide coalition focused on protecting West Virginians from vaccine-preventable diseases. Prior to the current project, the CRHD developed a publicly available video series answering COVID-19 vaccine questions in collaboration with the WV Joint Interagency Task Force, the WV Department of Health and Human Resources’ Bureau for Public Health, and the WVU Public Interest Communication Research Lab. The video topics were identified through a survey of West Virginians conducted by the WVU Public Interest Communications Research Lab (PIC Lab). Video topics were based on the most common questions/concerns that West Virginians had about COVID-19 vaccination; the PIC Lab was involved in drafting the video scripts.

### 2.2. Community Advisory Board

We successfully recruited and engaged 25 stakeholders from various regions to ensure that the educational interventions to be developed would be acceptable, feasible, and sustainable in local communities. The stakeholders guided our recruitment strategies, intervention delivery, and the development of a culturally and health literacy-appropriate educational intervention to increase COVID-19 knowledge and vaccine acceptance. By leveraging our prior collaboration, we built local capacity and community partnerships to reduce COVID-19 disparities. A statewide stakeholder advisory team guided the development of the educational training curriculum, recruitment of focus group participants to assess needs and gaps, and implementation strategies. The educational intervention focused on addressing misperceptions and barriers regarding COVID-19 vaccine development, safety, and side effects, as well as how to motivate patients for primary and secondary prevention measures, dispel myths, and provide accurate information for COVID-19 vaccine uptake.

### 2.3. Use of the Exploration, Preparation, Implementation, and Sustainment (EPIS) Framework

A systematic approach to mapping the planning and implementation of the COVID-19 educational intervention was utilized. Early in the development of the intervention, the project team explored the relevance of existing implementation frameworks, assessment tools, and resources for improving vaccine acceptance, especially within a West Virginian context, as it relates to Appalachian culture and health literacy. Specifically, an adapted version of the Exploration, Preparation, Implementation, Sustainment (EPIS) framework guided the intervention, as it is logical, evidence-based, and has been used in the design of public health interventions (the sustainment phase is excluded given that implementation of the intervention is still ongoing) [20].

**Figure 1:**
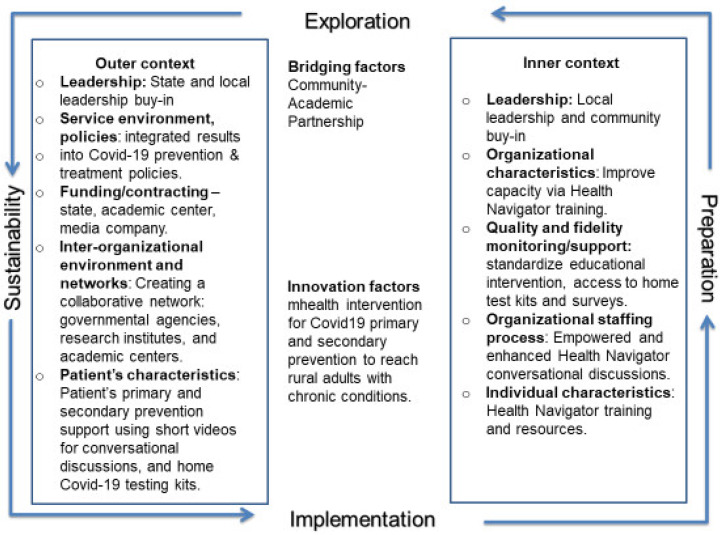
EPIS framework.

The EPIS framework describes four well-defined phases that guide the implementation process and success of intervention delivery. The environment for the outer context and the local culture, climate, and patient characteristics, knowledge, beliefs, vaccine uptake, and hesitancy barriers, or the “inner context” that impacted COVID-19 behavior and education, guided the team to implement the community-based educational intervention in rural communities (Figure 1).

The Exploration phase included semi-structured interviews that were conducted via telephone among 25 WV adults living with chronic diseases to explore their perceptions and beliefs regarding COVID-19 health literacy, risks, beliefs, and vaccination status. The majority of the interview participants were female (74%), did not have a college degree (54.5%), and were non-Hispanic whites (96%), representative of the state’s racial/ethnic characteristics. In addition, 36% of the interviewees reported an annual income of less than USD 50,000. Although the majority (87%) had received the recommended primary COVID-19 vaccine series, vaccine hesitancy and non-adherence were reported. Thirteen percent of the participants reported fear of potential vaccine side effects, concerns about vaccine safety, were against vaccinations in general, and believed that it would be better for nature to take its course for any infection. Considering interview participants were individuals with chronic conditions with a higher risk for severity of the infection if contracted, overall, participants were positive that the COVID-19 vaccination would protect them from serious illnesses, hospitalization, and death. In addition, the use of a community-based educational intervention to help improve knowledge and vaccine uptake was noted as a desire to learn more from reliable sources. Participants expressed ways to prevent and treat the infection and the need for trusted messengers to deliver the intervention.

Thematic analysis from the interviews also showed that participants’ health was impacted by COVID-19. More specifically, participants reported physical, mental, financial, and relationship challenges due to social isolation. In addition, brain fog, heart issues, fatigue, and mortality among family and friends due to COVID-19 were also reported. Perceived susceptibility and severity were high due to their pre-existing chronic conditions, and even with protection from vaccination and following masking guidelines. However, people were conflicted by the fear of COVID-19 and its impact on families and the benefits of vaccine uptake versus vaccine development concerns and its side effects, safety, freedom of choice, and government overreach. The interviews revealed key considerations for tailoring COVID-19 risk messaging among high-risk WV adults, such as needing succinct, clear information regarding potential long-term risks and consequences of COVID-19 infection, vaccine safety, and booster efficacy.

These findings led to the development of additional videos regarding COVID-19 risk and the importance of vaccination among those living with chronic disease, long COVID or post-COVID conditions, and the importance of keeping up to date with boosters to protect against variants of the virus that causes COVID-19.

In the Preparation phase, a team of public health and health communications experts developed, tested, and revised the curriculum for training HNs. The curriculum and educational materials were reviewed by experts and revised to tailor them to the local culture and health literacy level of rural adults and to develop patient-centered interventions. For example, a script and discussion flow chart for HNs to use to conduct the intervention was developed. During this phase, all materials to be used for the intervention and for training the HNs were presented to the CAB for review and were revised as needed. Once finalized, all WV HSTA-based HNs (high school students) were trained during a week-long “Biomed Camp” that was held in person on WVU’s health sciences campus. Similarly, WVU-based HNs (undergraduate and graduate students) were trained in person and via Zoom. HNs were successfully trained, and a supervision model was established. Long-standing community–academic partnerships between team members and the HSTA community prior to the launch of the project facilitated access to participants, mutual trust, and willingness to attend meetings, collaborate, and contribute to improving knowledge and practices. The technical support provided by the academic institution partners of WVU also played a critical role in addressing technology-related issues with tablets, videos, REDCap surveys, and the upload of educational intervention materials.

In the Implementation phase, HNs delivered the educational intervention in their local communities with close monitoring and supervision from the project team, WV HSTA program staff, and teachers. Matrices allowed for mapping the feasibility of recruiting an adequate number of stakeholders for the CAB, participants for semi-structured interviews, and potential participants for the intervention (WV adults living with chronic disease). Factors and context for exploring the EPIS framework for the engagement of the academic–community team in all four phases were evaluated, as well as the diversity and representativeness of the statewide CAB using meeting attendance, feedback on the training curriculum, and recruitment strategies for needs assessment interviews and intervention implementation. The fidelity of the HN-delivered educational intervention, using an adaptable script and flow chart to tailor participants’ concerns by watching brief educational videos of trusted WV healthcare providers, was also addressed.

### 2.4. COVID-19 Educational Intervention

**Figure 2:**
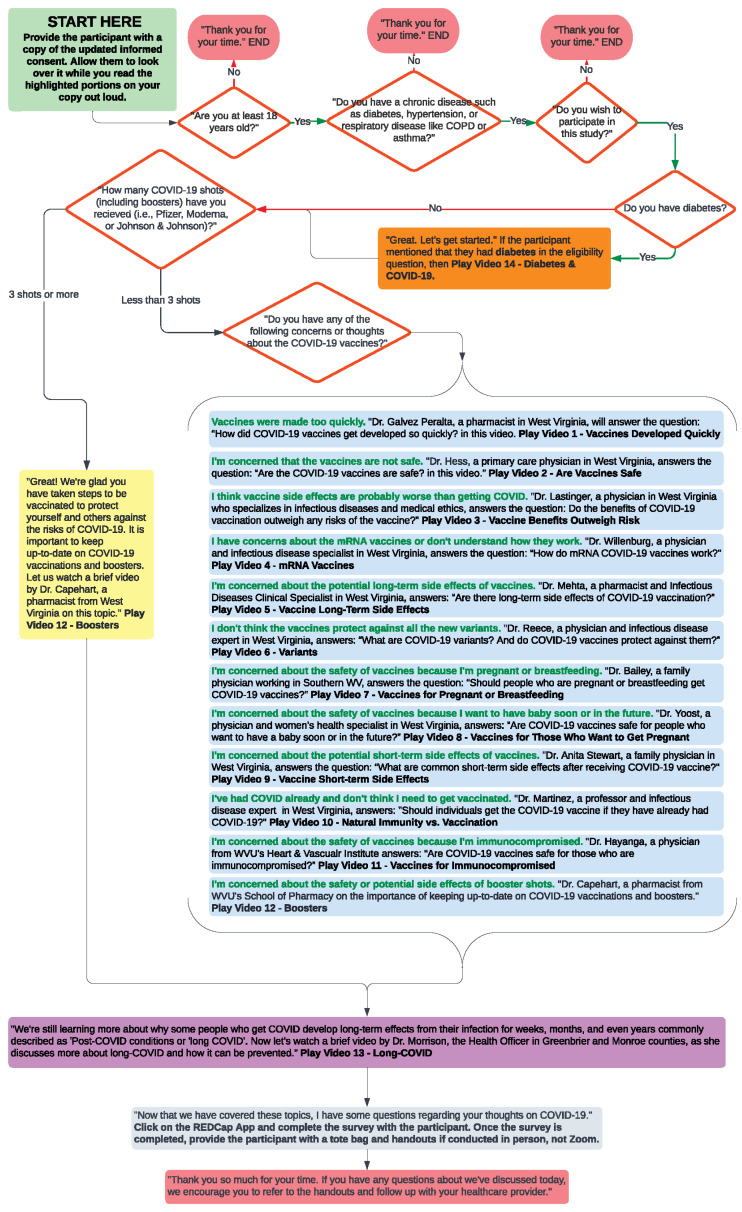
Educational intervention flow chart and scripts for Health Navigators.

The project used a post-only quasi-experimental study design for the educational intervention aimed at addressing concerns to improve COVID-19 knowledge and vaccine uptake among rural adults living with chronic disease(s). The educational intervention was culturally tailored and focused on addressing misperceptions and barriers regarding COVID-19 vaccine development, safety, and side effects, as well as how to motivate patients for primary and secondary prevention measures, dispel myths, and provide accurate information for COVID-19 vaccine uptake. Briefly, the intervention included the following components: (1) assessment of the chronic conditions and vaccination status to tailor the use of (2) three to four short educational videos (1–2 min each in length) developed using social and behavioral science insights. The videos were developed as part of a larger statewide campaign to address common COVID-19 vaccine risks, concerns and questions, and vaccination benefits. (3) Using a standardized script and discussion flowchart (Figure 2), the HNs guided participants in viewing specific videos by local WV health experts.

Lastly, (4) participants completed an online survey regarding their COVID-19 risk perceptions and vaccination beliefs and intentions.

### 2.5. Educational Materials and Videos

Video education has been shown to be effective in promoting self-care behaviors [21,22]. Video learning is effective as it helps to standardize the messaging and can be updated as needed, leaving more time for HNs for conversational discussions and engagement for vaccine uptake among rural adults with chronic conditions. These videos served to address concerns and information needs that were most evident for those in the contemplation stage regarding COVID-19 vaccination. Additionally, the videos aimed to provide information that could shape beliefs and move those who may have been in the pre-contemplation stage to contemplation [23]. We used 14 videos developed for the project or prior by the CRHD and PIC lab (see academic–community partnership section). Six new videos were developed for the project based on the qualitative interviews in the planning phase. Eight videos were also updated with new information. Activities for producing the new videos included the following: (1) CRHD selected and invited potential healthcare professionals to take part in the videos and establish a schedule of video shoots, (2) worked with the COVID-19 vaccination communication team to draft scripts for the videos using evidence-based information and adhering to the Center for Disease Control and Promotion (CDC)’s COVID-19 vaccine recommendations, (3) edited the videos for content and time (1–2 min), and (4) provided the final YouTube videos for streaming and made them available for download for an online platform and HN tablets. The new videos included: COVID-19 vaccination for people who have diabetes; the risk of long COVID and the role of vaccination in reducing risk; common side effects from COVID-19 vaccination; staying up to date on COVID-19 vaccination (primary series and boosters); COVID-19 vaccination for people who are immunocompromised; and immunity from disease (natural immunity) vs. immunity from vaccination.

### 2.6. Health Navigator Training, Fidelity, and Supervision

The HNs were recruited from both West Virginia University’s health-related undergraduate and graduate programs. Additionally, WV high school students were recruited from the WV HSTA program. All HNs completed a 16 h training on the COVID-19 determinants and epidemiology, health communication and how to talk to participants, decision making, and ethical dilemmas to address participant concerns. The training curriculum was programmed to culturally tailor the project using “conversational discussions” for knowledge transfer in community settings. The training helped to empower and enhance the skills of HNs using hands-on, mock interventions, fidelity checks, and use of training resources (e.g., flow chart, use of secure tablets with uploaded educational videos, educational handouts, and online REDCap survey). In addition, printouts of educational information, resources, and home COVID-19 testing kits were provided to HNs for distribution, as necessary.

The use of a community-based, locally trained HN model for educational intervention to address COVID-19 vaccine concerns and updates among rural adults with chronic conditions is not frequently used in the state. The extensive network of HNs in all counties of WV in the study served as a basis for community outreach and extension as proposed by the university’s lands grants mission. They served as trusted and “natural helpers” who served as health advocates for their communities. HNs received supervision and case management twice a month from the principal investigator and leadership team. Fidelity audits, using direct observation and a standardized checklist, were completed to ensure the educational intervention protocol was correctly followed and in the prescribed order and that modifications were incorporated as per protocol. All HNs completed a mock educational session with their peers. We considered 90% adherence to standardized fidelity checklists to be acceptable.

Supervision and support were provided by the expert trainer and principal investigator of the study, who monitored treatment fidelity throughout the project, aligning with behavior change recommendations. This ongoing oversight was crucial for ensuring the effectiveness of the intervention and the integrity of the research findings. Because coaching and feedback improve post-training efficiency, biweekly HN meetings provided supervision and problem-solving opportunities. Direct observations by the leadership team during training and mock sessions were completed in 100% of the training sessions for intervention fidelity. Observations included the use of the flow chart to ask questions and show educational videos tailored to the individual’s vaccine dose(s), medical history, concerns related to vaccine development, and long COVID. HNs provided comments to explain deviations from the curriculum. In addition to personnel observations, during some HN in-person visits to schools, churches, and other public places, teachers provided direct supervision to HNs during the first year of the study. In addition, the REDCap surveys were anonymous and were completed in a secure environment to improve recruitment and completion rates.

### 2.7. Participants

Participants were selected using a convenience sample of adults with chronic conditions screened for eligibility and willing to participate in the semi-structured interviews and educational intervention from November 2022 to October 2023. Eligibility included adults (18 years and older) with at least one chronic condition, willing to complete the intervention and post-survey assessment, and living in West Virginia. Exclusion criteria included individuals aged below 18 years with no chronic conditions, moderate to severe cognitive impairment or psychiatric disorder, and an inability to complete the educational intervention. HNs recruited individuals living with chronic disease from their local communities to participate in one-on-one discussions about COVID-19 risks and prevention. The use of this model allowed for bidirectional communication between communities and researchers to address COVID-19 health disparities and improve the health outcomes of high-risk patients. In addition, it was deemed innovative and resourceful to change and adapt to the growing demands for patient education and address mis/disinformation for individuals living with a chronic disease. This study was conducted according to ethical guidelines, and all procedures for this research study were approved by the Institutional Review Board at a large public university. All participants provided verbal informed consent prior to their participation. A subsample participated in semi-structured interviews to provide qualitative feedback about the needs and gaps for developing the COVID-19 training curriculum and educational intervention.

### 2.8. Data Collection Measures

Post-intervention survey data were collected, and HNs assisted with surveys as needed. The data collection tool was an online survey built using the Research Electronic Data Capture (REDCap) survey platform. The survey included 77 items encompassing a variety of topics, such as demographics, COVID-19 characteristics (e.g., have contracted it and/or been vaccinated with the primary series and/or boosters), perceived susceptibility, severity, long COVID susceptibility, long COVID severity, health history in terms of chronic conditions, social norms, vaccine concerns, vaccine intent, and self-efficacy. The survey was developed by the WV Joint Interagency Task Force [24], and data were collected using reliable and validated instruments by a senior team member. Participants either completed the online survey on the tablet provided by their HN or answered questions if they had lower computer or digital literacy. The present paper focuses on the vaccination status and concerns for COVID-19, as well as the willingness of unvaccinated participants to get vaccinated after the educational Intervention.

### 2.9. Data Management and Analysis

We analyzed data using SPSS version 29 (IBM, Armonk, NY, USA). Our a priori sample size calculation indicated an enrollment of 656 participants to provide 80% power to detect differences between groups based on vaccine status. Basic descriptive statistics and/or frequency analyses and correlational explorations were conducted for the study variables. Although missing data were present throughout the survey responses, they were handled using pairwise deletion to retain as much available data as possible. Furthermore, although missing data can adversely affect the accuracy of inferences made in inferential statistical tests, we had a large sample size (*n* = 1368) that may have circumvented this issue. The deidentified data were imported from REDCap into SPSS for cleaning and final analysis.

## 3. Results

Between November 2022 and October 2023, 45 HNs delivered an educational intervention in 52 out of the 55 counties in WV. This intervention reached a total of 1368 West Virginians residing in local communities (project implementation is still ongoing). Participants were mainly women (59.2%) and did not have a college degree (61.8%). The mean age was 43.5 ± 18.8 years and ranged from 18 to 90 years. Participants reported living with an average of 2.1 ± 1.4 chronic conditions (range 1–11). The prevalence of self-reported chronic diseases included diabetes (39%), hypertension (36.7%), mood-related disorders, such as anxiety and depression (29.1%), high cholesterol (20.8%), arthritis/osteoarthritis (19.1%), and respiratory diseases, such as asthma, COPD and chronic bronchitis (18%). More than half (56.5%) of the participants reported having two or more comorbid chronic diseases or multimorbidity. Regarding their vaccination status, 81.2% of participants reported receiving the primary vaccination series, and 63.1% received the primary vaccination series and at least one booster. However, 18% were unvaccinated or did not complete their primary vaccine series. Vaccine hesitancy did not differ by gender or age, but those with lower education and income had significantly higher vaccine hesitancy (*p* = 0.005).

The educational videos focused on improving COVID-19 health literacy. While all participants were shown the long COVID video, individuals who had completed the primary vaccine series viewed the importance of keeping up-to-date with vaccine boosters. For those without the primary vaccine series, videos were tailored to address their questions or concerns related to COVID-19 vaccines. Table 1 provides a breakdown of the videos and conversational discussions with participants.

The results suggest that there were more discussions related to how the COVID-19 vaccines were developed so quickly (22.4%), the importance of vaccination to protect variants (14.3%), and addressing concerns related to safety (22.3%). Educational discussions also focused on mRNA vaccines, short- and long-term side effects of vaccines (16.8 and 10.5%, respectively), and the importance of vaccine uptake for those who are immunocompromised (11%).

Results also demonstrated that participants with higher concerns were more likely to be unvaccinated and not complete their primary series or boosters (χ^2^ = 308.9; *p* < 0.001; Table 2). Participants who were unvaccinated or did not complete their primary vaccine series had the most concern about the vaccin’s potential side effects and safety. In general, these participants were against vaccination and considered sickness and death from COVID-19 a natual occurrence. However, the educational intervention improved the willingness of participants who were either unvaccinated or did not complete their primary vaccine series (11.4%).

### Home Monitoring of COVID-19 Testing

This study also expanded opportunities for home COVID-19 self-monitoring by providing HNs with home test kits to distribute among high-risk individuals. Two hundred and fifty home test kits were distributed to participants who requested them as they were hesitant to go to the hospital emergency room, healthcare providers, or health departments when they experienced symptoms or were exposed to others who had tested positive for COVID-19. Studies have documented the value of home monitoring for chronic diseases and medication adherence [25]. In recent studies, the effective integration of home test kit monitoring showed that patients were satisfied with self-monitoring for a successful intervention [26].

## 4. Discussion

This paper presents the results from a community-based educational intervention to improve vaccine uptake among rural patients with self-reported chronic diseases. The findings revealed that more than half of the participants had multimorbidity, i.e., two or more chronic conditions. We also found that individuals with comorbid chronic diseases had significantly greater vaccine uptake as they perceived their disease status increased their risk and severity for COVID-19 infection; this concurs with prior research that shows chronic disease patients are associated with worse COVID-19 clinical outcomes and are willing to adopt individual risk management behaviors [27]. The majority of the participants reported having the basic primary vaccine series and/or had at least one booster. Yet it was concerning that one-fifth of participants’ risk perceptions were not associated with their medical diagnosis and remained vaccine-hesitant. If specific individuals continue to be vaccine-hesitant, primary care and medical specialty groups could focus on patient engagement for vaccine uptake during clinic visits. Indeed, research has shown that lower vaccine literacy could be germane to lower socioeconomic status in rural areas where patients are impacted by a variety of social determinants of health [28].

The demographics of the vaccine-hesitant participants showed they were more likely to have lower education and income than those with vaccine uptake; this may likely be due to distrust and misinformation, an inability to understand the recommendations of public health agencies, a lack of access to broadband and internet capabilities or a lack of digital literacy and information that could have led to their belief that the vaccine is unsafe, has potential side effects, and that natural immunity is better than acquired immunity through vaccination. This highlights the importance of risk messaging in rural communities that provide a basis for tailoring educational strategies and communication of public health messages based on patient health literacy, belief, and vaccine status. Larson et al. [29] noted that trust in vaccines is a multidimensional construct impacted by trust in the healthcare system, science, and government. It also exists in the context of the broader societal trust related to cultural and economic capital (such as education and income). Hence, future research should explore the various factors that impact vaccine-related trust in rural patients with chronic diseases.

Limited studies have focused on rural patients, and even fewer studies have explored educational intervention to enhance vaccine uptake among rural adults with chronic conditions and multimorbidity in community settings [30]. Hence, this study adds to the body of knowledge. The EPIS [31] framework was useful as it allowed for the team to have early planning and complete needs and gaps analysis to address challenges and resolve issues for a culturally tailored intervention. In addition, using a novel, community-based local HN model with a supervision/fidelity management approach allowed for reaching the hard-to-reach rural patients with chronic diseases; this is particularly important as COVID-19 variants continue to impact individuals and communities. Hence, the use of locally trained HNs can be especially pertinent for sustainable educational interventions and risk messaging as they live and are trusted individuals in rural communities. These trained staff helped in the outreach efforts of this project to educate their family members, neighbors, church members, and others. The community-based educational intervention also addressed the Appalachian culture of mistrust or distrust of institutions and involved resource sharing (e.g., home test kits) with patients who were hesitant to go to a clinic or health department. Our findings suggest that rural patients were open to receiving COVID-19-related information through video education by local experts and having a conversational discussion with HNs about vaccine uptake. Future studies should compare this strategy to other educational strategies in order to prevent people from looking for information on non-trustworthy sites, reduce misinformation, and improve vaccine acceptance. While the project is currently ongoing, utilization of our academic community infrastructures, such as the HSTA, that were already in place in 27 counties of the state, supported the implementation delivery in the majority of counties.

The use of the EPIS framework was useful to validate the key elements of our academic–community collaboration [32]. In addition, it demonstrated relevance and usefulness in recording our prior community-engaged research projects’ partnership as well as in planning and implementing a promising, cost-effective intervention targeting rural adults [20]. While a prior systematic review of the framework primarily emphasized the implementation phase [33], we used all four phases to reflect our challenges and ensure resilience during changing local contexts, particularly in view of the COVID-19 vaccine policies and resources available in the state. Currently, no project has focused on adults with chronic conditions or used HN-delivered COVID-19 educational intervention to improve vaccine uptake in the Appalachian context. Hence, our study offers new insights into our understanding of an important yet understudied public health problem, In addition, by using the EPIS framework, the study provided a novel cost-effective approach for accessing an unique rural population in a resource-poor setting.

The pandemic has disproportionately impacted rural states such as West Virginia, where there is a higher burden of chronic disease and multimorbidity (e.g., diabetes, hypertension, and COPD), which are risk factors for severe COVID-19 and long COVID [34]. Hence, our community-based educational intervention and partnership is an example of an innovative way for academic and community partners to successfully address and support knowledge gaps and rural health disparities. The expertise and collaboration of team members were critical not only to develop and implement the intervention but also simultaneously to promote local capacity to deliver evidence-based interventions in all counties of WV. These partnerships can also be useful for providing experiential learning opportunities and training the next generation of public health workers to be prepared to address emerging health issues, such as the COVID-19 pandemic. This study showed that community interventions using trained HNs are feasible and acceptable and could be a successful model for educating high-risk individuals and helping connect with healthcare services for promising best practices [20]. It also symbiotically introduced and educated trusted local messengers to public health practice.

In addition to the scientific knowledge gathered from this project, it also teaches important lessons for the future implementation of community-based interventions in rural Appalachian states. For example, the planning and exploration phase was critical to our engagement with communities of need that involved stakeholder feedback for tailoring and shared resources and decision making for developing successful approaches. Leveraging our prior collaboration and long-term relationships with the HSTA communities, personnel and teachers also played a vital role in forming a cohesive team that helped guide the project activities in all four EPIS phases. Importantly, we enlisted the perspectives of healthcare providers, service organizations, faith-based institutions, formal and informal community leaders, and patients to inform implementation. Since the preparation phase directly promoted successful knowledge transfer and vaccine uptake, identifying a feasible and culturally acceptable approach to project implementation was important to our success [35]. During the exploration phase, researchers and practitioners also worked together to identify the training curriculum content and culturally tailor the project implementation components. For example, the use of “conversational discussions” for knowledge transfer in community settings was deemed to improve non-judgmental knowledge transfer without impeding fidelity [36]. Finally, this study validates the importance of community–academic partnerships to bridge interlinkages to inform implementation in all counties in the state. The successful recruitment of 1366 rural patients in a 12-month time frame revealed promising effectiveness with multiple recruitment strategies.

Many positive aspects are noted for the innovative academic–community partnership (e.g., positive attitudes of the team, HNs, staff, COVID-19 education, feasibility and acceptability, and participation of a larger number of rural high-risk adults). The use of trained HNs also supports our previous behavioral interventions so that non-professionals without prior experience can be trained to deliver health interventions with fidelity and careful monitoring [37]. Reliance on low-cost educational strategies, such as community health workers delivering health education and related services, continues to grow to address the shortage of health workers in rural states. Yet, the impact of using this novel approach on the public health education workforce is not well understood in West Virginia. Our findings have implications for improving vaccine uptake and dispelling myths and misinformation. Educating rural patients with accurate COVID-19 information, especially regarding their vaccine concerns, in ways understandable to individuals from a variety of socioeconomic and educational backgrounds can improve vaccine literacy and uptake [38]. In addition, tailored, easy-to-understand health communications delivered through a variety of different modalities may allow vaccine-hesitant individuals with chronic diseases to make more informed health decisions and increase their willingness to receive the COVID-19 vaccine.

The present study is, however, not without limitations. The educational intervention used a quasi-experimental design, and the assessment was conducted at a single point in time. Furthermore, we acknowledge possible bias for socially desirable responses for participants who completed the online survey in front of their HNs or were assisted for the completion by those (based on their preference) with a lower computer and digital literacy. As the pandemic continues to evolve, longitudinal methods of data collection and experimental designs should be considered to examine the impact of educational interventions on improving vaccine acceptance and COVID-19 health literacy, which may change over time. The HNs were asked to target rural and underserved individuals from their communities for the surveys. No attempt was made to determine the educational level, employment, income, or political affiliation of participants. Finally, mixed methods studies with qualitative and quantitative assessments can provide a better understanding of various factors associated with vaccine hesitancy.

## 5. Conclusions

This study provides insights into the need for tailored educational interventions based on disease status, which has implications for public health and patient care in rural and underserved communities. The use of the EPIS framework and its phases provided useful guidance in the development and planning of interventions and strategies in rural areas. Academic–community partnerships can be useful for successful knowledge transfer for vaccine acceptance to reduce rural health disparities. These partnerships are also useful for providing experiential learning opportunities and training the next generation of public health workers to be prepared to address emerging health issues such as the COVID-19 pandemic.

## Figures and Tables

**Table 1:** Participant-Selected COVID-19 Video Frequencies.

COVID-19 Frequently Asked Questions Videos Selected by Participants	*n*	%
Why is vaccination so important for people who are immunocompromised?	150	11.0
How do mRNA COVID-19 vaccines work?	141	10.3
What are the common short-term side effects of COVID-19 vaccination?	143	10.5
Are there long-term side effects of COVID-19 vaccination?	230	16.8
What are COVID-19 variants? And do COVID-19 vaccines protect against them?	196	14.3
Should individuals get the COVID-19 vaccine if they have already had COVID-19?	136	9.9
How did the COVID-19 vaccines get developed so quickly?	307	22.4
Are the COVID-19 vaccines safe?	305	22.3
Do the benefits of COVID-19 vaccination outweigh any risks?	187	13.7
Are COVID-19 vaccines safe for people who want to have a baby soon or in the future?	106	7.7
Should people who are pregnant or breastfeeding get vaccinated?	102	7.5

**Table 2:** Associations of COVID-19 vaccination status with vaccine concerns among adults with chronic conditions (*n* = 1368).

	Vaccine Concern (%)		*p*-Value	
	No Concern	One or more concerns		
COVID-19 vaccination status				
2 shots of Pfizer/Moderna or 1 shot of Johnson & Johnson, plus GOT 2 booster shots	83.60%	16.40%	<0.001	***
2 shots of Pfizer/Moderna or 1 shot of Johnson & Johnson, plus GOT a booster shot	59.20%	40.80%		
2 shots of Pfizer/Moderna or one shot of Johnson & Johnson, but have NOT gotten a booster shot	33.60%	66.40%		
1 shot of Pfizer/Moderna	19.20%	80.80%		
Not vaccinated (no COVID-19 vaccine doses received)	16.50%	83.50%		

*** indicates *p* < 0.001.

## Data Availability

Data are contained within the article.
